# Survey Data on Attitudes Towards Foreign Aid & Development in France, Germany, Great Britain, and the U.S.

**DOI:** 10.1038/s41597-025-05135-0

**Published:** 2025-07-01

**Authors:** Felipe Torres-Raposo, Soomin Oh, Paolo Morini, Jennifer Hudson, David Hudson

**Affiliations:** 1 London School of Economics and Political Science, Government Department, London, WC2A 2AZ United Kingdom; 2 University of Birmingham, International Development Department, Birmingham, B15 2TT United Kingdom; 3 King’s College London, Policy Institute, London, WC2B 6LE United Kingdom; 4 University College London, Department of Political Science, London, WC1H 9QU United Kingdom

**Keywords:** Politics, Interdisciplinary studies, Communication

## Abstract

Public support is crucial for shaping effective foreign aid policy and development cooperation. The Development Engagement Lab (DEL) has conducted surveys in France, Germany, Great Britain, and the United States to track and analyse public attitudes towards foreign aid and engagement with sustainable development. This data descriptor presents multiple datasets, identifying shifts in public opinion and behaviours, alongside underlying mechanisms explaining these attitudes and actions. The dataset comprises nationally representative panel data (2019 - 2024), repeated cross-sections (2019 - 2024), and several foreign aid subject-specific datasets. The DEL data contains 91 unique datasets, with 270,829 observations from 130,286 unique respondents. We have developed an R package, *DELdata*, to facilitate the use and dissemination of these datasets. These data will enable scholars, practitioners, and policymakers to produce valuable insights that will generate a better understanding of how citizens think about, and engage with, foreign aid and sustainable development.

## Background & Summary

Public support is essential for the quantity and quality of foreign aid spending, development policy, programming, and international cooperation. Although foreign aid is financed by the public purse through taxpayer contributions, and is subject to government approval and oversight, aid is spent in support of non-taxpaying citizens of other countries. This tension has been recognised by both policymakers and academics^1–4^, who have long argued that public support allows political elites to operate more effectively within a contested political space. Direct action from the public—including through donations, activism, or volunteering—supports and legitimises the work of international development NGOs, who can advocate for further government efforts, or who deliver aid programmes to developing countries directly^5,6^.

As we approach the 2030 Sustainable Development Goals deadline, citizens’ understanding of, and engagement with development efforts, will be a critical part of addressing global issues such as ending extreme poverty or achieving gender equality worldwide. Contemporary global and national events such as the cost of living crisis, the climate crisis, humanitarian refugee crises, and international conflict are shaping people’s attitudes towards helping distant strangers in different ways. On the one hand, we observe increases in charitable donations in some countries^7^, concomitantly, we have also seen public support for development cooperation fall significantly^8^.

The Development Engagement Lab (DEL) dataset is a publicly available source of longitudinal and cross-sectional data on key donor countries’ public attitudes and engagement with global poverty. We collected these data through a series of dedicated surveys and experiments described below. A key feature of the data is the multidimensional measures of key indicators, for example, measuring the general concept of support for development as a set of attitudinal indicators ranging from ‘concern about levels of poverty in poor countries’ to ‘support for current levels or increases in aid expenditures’, and to ‘support for giving aid in the national interest or for altruistic reasons’.

Beyond public attitudes, the data also allow for exploring and understanding public engagement with global poverty and development through respondents’ self-reported behaviours. The data include ten key indicators capturing actions the public take to tackle global poverty, lobby government, or support international NGOs. This battery is used to segment respondents into six groups: Negatively Engaged, Totally Disengaged, Marginally Engaged, Transactionally Engaged, Purposively Engaged, and Fully Engaged^9^. This audience segmentation helps us understand the characteristics of individuals who take specific actions over others, how to communicate with them, and what ‘drives’ their actions.

This collection of data broadens the scope of research in public engagement with global poverty and sustainable development both methodologically and substantively. Methodologically, the Development Engagement Lab team has made use of a range of experimental approaches including survey, conjoint, and list experiments aimed at identifying people’s preferences, beliefs, heuristics, and knowledge around a wide range of development issues and the factors that affect these. Substantively, the datasets provide deep dives into a broader set of thematic issues of importance for global development. Some issues covered through ad hoc surveys and experiments include public perceptions and engagement with climate change, COVID-19, global gender (in)equality and feminist development policy, and global conflicts, including the war in Ukraine.

We expect researchers in the fields of public opinion, foreign aid, and sustainable development will be able to take advantage of the granularity and richness of the data to shed new light on citizens’ preferences, attitudes, and behaviours towards global challenges, and towards other issues related to development cooperation. Key advantages of the datasets are the large sample sizes and the panel structure. The sample sizes allow for sufficiently-powered sub-group analysis of specific population segments and monitoring changes over time, as well as allowing researchers to leverage the temporal structure of the data using mixed effects, dynamic, and latent class models. This is helpful not only for researchers to understand what drives changes in public opinion and engagement, but also for development NGOs and charities who need deep insights to target key audiences and drive change.

## Methods

The DEL project was approved by University College London’s Department of Political Science’s Ethics Committee (15 September 2018, approval number 550710) Table 1.

**Table 1:** Dataset Descriptions.

Survey Type	DOI	Description
Panel	10.7910/DVN/0AE1H2	DEL Panel 2019: Annual longitudinal public opinion survey and data on global poverty and development from 2019^23^
	10.7910/DVN/KWTWCF	DEL Panel 2020: Annual longitudinal public opinion survey and data on global poverty and development from 2020^24^
	10.7910/DVN/CCUZYI	DEL Panel 2021: Annual longitudinal public opinion survey and data on global poverty and development from 2021^25^
	10.7910/DVN/WZPNNU	DEL Panel 2022: Annual longitudinal public opinion survey and data on global poverty and development from 2022^26^
	10.7910/DVN/FTRXFO	DEL Panel 2023: Annual longitudinal public opinion survey and data on global poverty and development from 2023^27^
	10.7910/DVN/PT7REY	DEL Panel 2024: Annual longitudinal public opinion survey and data on global poverty and development from 2024^28^
Tracker	10.7910/DVN/L9DUKN	DEL Tracker 2020: Cross-sectional biannual public opinion survey and data from 2020^33^
	10.7910/DVN/PEH21C	DEL Tracker 2021: Cross-sectional biannual public opinion survey and data from 2021^34^
	10.7910/DVN/L2STP0	DEL Tracker 2022: Cross-sectional biannual public opinion survey and data from 2022^35^
	10.7910/DVN/XH4K0E	DEL Tracker 2023: Cross-sectional biannual public opinion survey and data from 2023^36^
	10.7910/DVN/HU6UFY	DEL Tracker 2024: Cross-sectional biannual public opinion survey and data from 2024^37^
Sandbox	10.7910/DVN/4OV6AH	DEL Sandbox 2019: Responsive public opinion survey and data from 2019^38^ (Topics vary by year and country - See Table 13)
	10.7910/DVN/LLM8VW	DEL Sandbox 2020: Responsive public opinion survey and data from 2020^39^ (Topics vary by year and country - See Table 13)
	10.7910/DVN/DUUPRY	DEL Sandbox 2021: Responsive public opinion survey and data from 2021^40^ (Topics vary by year and country - See Table 13)
	10.7910/DVN/ELT7M0	DEL Sandbox 2022: Responsive public opinion survey and data from 2022^41^ (Topics vary by year and country - See Table 13)
	10.7910/DVN/XZ1XUZ	DEL Sandbox 2023: Responsive public opinion survey and data from 2023^42^ (Topics vary by year and country - See Table 13)
Script	10.7910/DVN/MBY5GD	R script to clean and harmonise the datasets^46^
	10.7910/DVN/NVJEZM	R script to create engagement-based DEL segmentation^47^

This database contains data from 91 online surveys in France, Germany, Great Britain, and the United States, collecting 270,829 observations from 130,286 unique respondents. Table 2 in the appendix provides additional information on the data collection for three types of surveys: *Panel*, *Tracker*, and *Sandbox*. In what follows, we provide a detailed explanation of the survey methodology and the three surveys.

### Survey Methodology

#### Sampling

The data for this study were collected using YouGov’s online panel. Third-party research institutions^10^ have highlighted the overall good performance of YouGov’s panel in multiple dimensions, such as the accuracy of multivariate relationships and overall variability across samples. YouGov’s online panel covers 2.5 million respondents in Great Britain, 5.2 million in the U.S., 780,000 in France, and 800,000 in Germany.

The data were collected from a target population consisting of adults aged 18 and over residing in Great Britain, Germany, France, and the United States. These countries are among the largest aid donor countries (as measured by aid volume) in OECD Development Assistance Committee (DAC) from 2019 until 2024^11^. Respondents from the panel were invited to participate via email and were unaware of the survey topic prior to participation, minimising self-selection bias. To encourage participation, respondents received incentives in line with YouGov’s standard panel engagement practices, such as points redeemable for rewards. Response rates for the study were typical of YouGov’s panel surveys, with wave-on-wave retention rates ranging from 70% to 80% depending on the sample demographics and elapsed time between survey invitations.

The sampling procedure employed a non-probability approach, utilising targeted quota sampling to ensure representativeness across key demographic characteristics, such as age, gender, education level, and political interests. Quotas were based on benchmarks from national censuses and official mid-year population estimates, ensuring alignment with the broader population. YouGov employs a dynamic ‘active sampling’ process, which adjusts respondent invitations in real time to fill quota targets, maximising alignment with the target population.

All participants provided informed consent at the time of joining the panel. This consent includes agreement for their data to be collected, stored, and used for research purposes, including sharing anonymised data with third parties. YouGov’s procedures are outlined in their Privacy Policy and Terms and Conditions. Participants were invited to take part in the study via email and were unaware of the survey topic before participation. No sensitive personal data were collected, and the datasets made available for this study are fully anonymised and do not contain any identifying information.

*Weighting Procedure* Post-stratification weights were calculated to calibrate for potential imbalances in the sample. Survey weights were calculated using an iterative proportional fitting (raking) process to adjust the sample distributions to match population benchmarks derived from national censuses and other authoritative sources. These weights correct for demographic imbalances in the sample, addressing biases related to panel recruitment and non-response. Specifically, weights were applied for variables such as age, gender, education, and political interest, ensuring that the weighted data align with the population distributions.

Given the sampling method and the weighting scheme we described, the overall approach yields representative estimates of the population within and across countries over time. Our data provider continuously updated sampling frames, reflecting the latest national elections and other demographic targets, and to help account for key trends and population dynamics, ensuring more reliable cross-country comparisons and more representative samples.

We also took several pre-survey harmonisation measures to ensure cross-country comparability over time, including rolling out both panel and repeated-cross sectionals within the same fieldwork windows in all four countries. This procedure ensured that the data captured common trends that affected all countries simultaneously, mindful that country-specific events or challenges can still affect specific countries. We conducted a Multi-group Confirmatory Factor Analysis across countries and waves to test for measurement invariance for a small set of core items. Table 3 in the appendix reports the results of Configural, Metric, Scalar and Strict models using five core items in our national surveys to measure individuals’ underlying concern with poverty and development issues. The results suggest no measurement invariance within countries across waves, while the evidence is weaker once we compare across countries. Researchers can expand on these tests, leveraging the extensive literature^12–14^ and codebase to perform these tests^15–17^.

### Survey design and harmonisation

To ensure a high degree of cross-linguistic and cross-country comparability, and to improve the quality of data collected, we take measures to maximise both interpretive and procedural equivalence^18^. First, the core survey questionnaires for data collection were initially developed in British English, with a key set of cross-sectional tracking and panel questions remaining fixed for the duration of the study, minimising potential measurement inconsistencies. This was translated by qualified professionals at YouGov and in partnership with DEL’s country consultants to maximise shared meaning across fieldwork countries.

Specifically, in Germany, translations were handled initially by YouGov’s translators, followed by verification by German consultants for linguistic, semantic, and technical accuracy. In France, French consultants took the lead for translation, with YouGov providing an additional technical validation check. Finally, for the U.S. surveys, the DEL team adapted the questionnaire to match American terminology and writing conventions. In all countries, we also ran pre-fieldwork script testing with country consultants, and where possible, used survey experiments to gather evidence to inform our choices between multiple alternative wording or framing options.

Second, we took measures to improve procedural equivalence^19^ by taking into account that response approaches vary across countries and cultures. We sought to use multiple items to measure key constructs, a variety of wording and framing approaches, and multi-item response scales (both five and ten points) for attitudinal questions^20^. Where appropriate, we randomised item order within question batteries, including when asking respondents to rank different options, and we systematically aimed to ease cognitively demanding question batteries by limiting the number of items and response options.

We simplified the wording and length of the surveys to reduce survey fatigue and avoided including double-barrelled, vague, or all-or-nothing types of questions^21^. For unipolar and bipolar items, we consistently used a small but sufficient number of categories, still obtaining sufficient variation in respondents’ answers^22^. Cross-survey contamination between different data collection types, detailed below, was also low: of 40,605 respondents that answered *Trackers* across the four countries, only 8,561 were also included in the *Panels*. We provide further detail about the different quality controls we underwent in the technical validation section of this paper, but first we outline the three data types included in the dataset.

### Panel

**Fig. 1:**
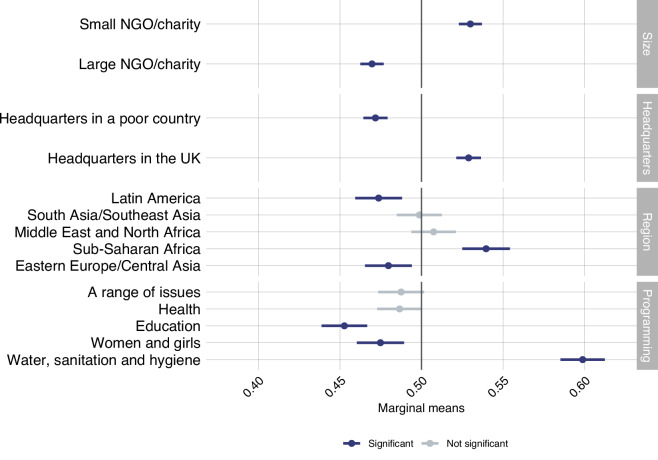
Factors affecting preferred aid project: results from a forced-choice conjoint experiment. Note: This figure shows the results of a forced-choice conjoint experiment. The dependent variable is measured as the marginal means of all levels within each attribute. This estimate conveys the favourability level toward profiles with a particular feature level, ignoring all other features. This experiment included four attributes: *Size, Headquarters, Region* and *Programming*. Respondents were presented with two NGO profiles and asked which NGO were more likely to donate. The *Size* attribute contains two levels that capture the size of the NGO: “Small NGO/charity” and “Large NGO/Charity”. The *Headquarters* attribute contains two levels, which report the location of the Headquarters of the NGO/Charity: “Headquarters in a poor country” and “Headquarters in the UK”. The *Region* attributes captures the regional focus of the NGO, including five different geo-cultural/geo-political regional classifications: “Latin America”, “South Asia/Southeast Asia”, “Middle East and North Africa”, “Sub-Saharan Africa”, and “Eastern Europe/Central Asia”. The final attribute is *Programming*, which captures the issues an NGO worked on. We included five levels: “A range of issues”, “Health”, “Education”, “Women and girls”, and “Water, sanitation, and hygiene”. The data of this conjoint experiment is available on the panel data Wave 3, only for Great Britain. The exact wording of the question and response options are in the appendix.

To capture changes in attitudes and behaviour over time as well as key mediators, we developed a panel survey including 47 fixed questions and a varying number of context-specific questions. We collected panel survey data annually between 2019 and 2024 in each of the four countries, yielding 24 panel datasets^23–28^. The fixed questions section includes an extensive battery of items aimed at eliciting, among many issues, levels of individuals’ support for aid, self-reported charitable donations to international NGOs, perceptions of the costs and benefits of aid, attitudes toward migration, and questions on economic outlook and trust. Table 4 in the appendix provides a breakdown of all the questions in the *Panel*. The year-specific questions section contains a bespoke battery of items responsively developed in collaboration between the DEL research team and the project’s stakeholders – over 40 international development NGOs and government ministries. For instance, in the 2023 survey^27^, we embedded a conjoint experiment to get a more complete understanding of which aid project characteristics (including purpose and region, to name a few) matter more for public support. In 2021, we explored how NGOs’ characteristics (size of the NGO, location of headquarters, and issue focus) affect respondents’ willingness to donate^25^. The results of this conjoint experiment are shown in Fig. 1.

The sample sizes ranged from at least 6,000 observations in France, Germany, and the United States to at least 8,000 in Great Britain. We systematically collected all *Panel* waves between September and October/November of each year. In Table 5 in the appendix, we summarise the data collection dates, sample size, and retention rates of the data. All surveys include survey weights to yield nationally representative samples. Table 6 in the appendix provides a visual metric of the depth of the topics covered in each country and wave.

*Panel* is an unbalanced panel where, on average, across all countries, the retention of first wave respondents gradually decreases from 70% in wave two, to 38% in the sixth and final wave. Samples were topped up with new respondents to keep sample sizes consistent in size across each wave. Table 5 in the appendix shows the aggregate retention rate pooling all countries together. Looking at each country individually, we find similar retention rates, except for Great Britain, where dropout rates are considerably lower than the other three countries. Table 7 in the appendix reports the retention rates between two subsequent waves by country. This level of attrition falls within the expected dropout rates in longitudinal studies that range from 30 to 70%^29,30^. Table 8 in the appendix summarises some key demographics included in *Panel*.

We explored whether there are systematic differences between the observed sample and attriters on their baseline (wave 1) characteristics exist. Table 9 in the appendix reports these two groups’ mean and standard deviations. We identified attriters as more likely to be younger, left-leaning males with higher incomes. There are no clear patterns regarding educational attainment (university or college education), as it varies wave by wave. We also compared *partial completers* who are respondents who took part in at least 2, but no more than 3 waves, versus *completers* who are respondents who participated in all waves, and the patterns are similar, *partial completers* are younger, are disproportionally male, attained lower levels of education, more left-leaning and earn less, vis-a-vis *completers*. We defer to researchers to determine the appropriate methods for addressing issues related to attrition. Several approaches have been put forward to deal with attrition in longitudinal studies, such as multiple imputation methods^31^, or leveraging advances in machine learning methods to deal with attrition^32^.

### Tracker

**Fig. 2:**
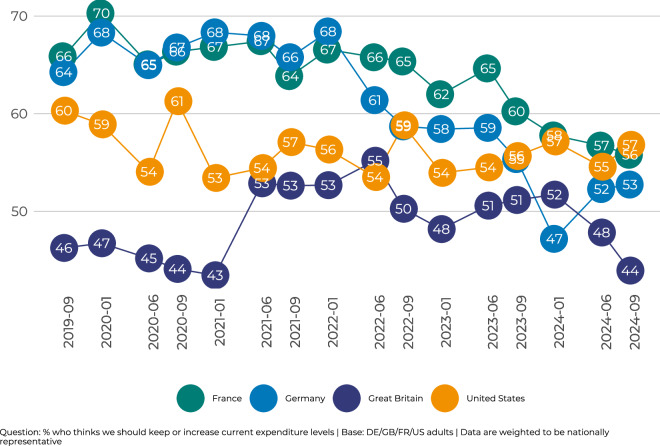
Trends in Support for Foreign Aid. Note: This figure shows the aggregate average level of support for foreign aid in all four countries. We measured support for overseas aid based on whether respondents wanted to maintain or increase the money their government spends on overseas aid. The dependent variable (y-axis) shows the share of respondents who want to maintain or increase government spending on foreign aid relative to the whole sample (including respondents who answered that they prefer not to say or Don’t know). On the x-axis is the month/year the survey was conducted. Estimates are weighted to be nationally representative. The exact wording of the question and response options are in the appendix.

In addition to the annual longitudinal panel survey, we collected two smaller repeated cross-sectional samples each year, capturing key attitudinal and behavioural indicators^33–37^. These data aim to identify and highlight changes in public attitudes and engagement with higher frequency, especially around key events and changes in the political, economic, and social landscapes. *Tracker* data were collected through a reduced 20-question survey drawn from the larger *Panel* instrument (except in the U.S., where a smaller core battery of 10 questions was collected), implemented in all four countries from 2019 until 2024 (10 waves). This means that for these 10-20 items, we effectively have three data points per year (two *Trackers* + one *Panel*). The sample sizes *Tracker* surveys in Germany, France, and the U.S. include at least 1,000 responses, with larger samples of 2,000 respondents available for some samples in Great Britain. Table 10 in the appendix summarises details of these samples, including collection dates and sample sizes. Figure 2 illustrates how *Panel* and *Trackers* datasets can be merged into a single file to examine the aggregate long-term trends in levels of public support for aid across countries. This figure shows the percentage of respondents who say they would like their government to keep or increase the current levels of development aid expenditure. Table 11 in the appendix summarises some key demographics included in *Tracker*.

### Sandbox

*Sandboxes* are the responsive component of the DEL project; an isolated testing environment outside the *Panels*. *Sandboxes* are deep dives into specific topics of interest, identified and developed in collaboration with project stakeholders across the four countries. Throughout the project, we conducted 27 surveys examining topics ranging from citizens’ beliefs and responses to the Syrian refugee crisis to global gender (in)equality^38–42^. The frequency of *Sandboxes* varied from year to year and country, driven by research priorities. For example, we conducted nine *Sandboxes* in France and five in the U.S. Most *Sandboxes* include a minimum and typical sample size of 2,000 responses, but for specific studies, this is much larger, up to 5,591 for a U.S. sample. Table 12 in the appendix provides information on the data collection dates, sample sizes of these surveys, and themes covered.

The *Sandbox* surveys also contain experimental designs to investigate citizens’ attitudes towards global challenges, including but not limited to humanitarian crises, war and conflict, and climate change. For example, in 2022, we studied how citizens in Great Britain engaged with the conflict in Ukraine, seeking to understand, among other key questions, whether respondents were willing to reallocate financial aid and donations from humanitarian crises in Africa and the Middle East to support Ukraine^41^. In 2021, we looked at U.S. citizens’ engagement with security as a key question for international cooperation, including public support for withdrawing their military forces from Afghanistan, and whether the public deemed the primary role of foreign aid spending as an important instrument to promote global stability^40^. In Table 13 in the appendix we provide further information on the topics covered in these surveys Table 14.

We also frequently used *Sandboxes* to test the effectiveness of different messages and forms of communication with the public, generating evidence informing charities’, NGOs’ and governments’ campaigning, education, engagement efforts. In these surveys, we investigated the role of different frames and narratives and how their impact on support for development actors or policies. For instance, in 2023, we tested how different frames of gender equality affected public support for feminist development policy in all four countries^42^.

## Data Records

The datasets associated with this work are available at the Harvard Dataverse repository^23–28,33–42^. These include data collected across different types of studies (tracker, panel, and sandbox) conducted between 2019-2024. Table 1 below outlines each dataset, including the corresponding DOI, and a brief description of the content (survey type, content, reference number).

All datasets include a unique respondent identifier, enabling users to link individuals across waves or merge data from different survey types. The data are available in both .sav and .csv formats, with each dataset structured by rows (respondents) and columns (variables). Column headers correspond to variable names (e.g., DELseg, track1), which are applied consistently across survey waves and formats.

Variable names follow a unified naming convention across countries, years, and survey types. Standard demographic variables measured using the same scale are harmonised, and variable names are designed to indicate the conceptual grouping or battery they belong to.

Each dataset is accompanied by a codebook and plain text version of the survey questionnaire. The codebooks describe each variable’s name, question wording, response values and labels, and any special coding (e.g., ‘don’t know’ or ‘prefer not to respond’). The questionnaires provide full wording, coding protocols, and, where relevant, details on randomisation or split-sample design.

The datasets are hosted on the Harvard Dataverse: https://dataverse.harvard.edu/dataverse/devengagement, organised into three Dataverses reflecting the survey types: Panels, Trackers, and Sandboxes. Within each Dataverse, subfolders are named using a consistent convention that includes the survey type and year of data collection. File names for datasets, codebooks, questionnaires, and data tables also specify the country, year, and, where applicable, survey wave (e.g., DEL_Panel_US_Wave_6_codebook.xlsx). Each dataset entry includes a persistent DOI, version history, and recommended citation to support transparency and reproducibility.

## Technical Validation

We employed several strategies to validate the data throughout the survey design and data collection life cycle. Before rolling out the surveys, the research team and country consultants inspected the questionnaires using testing links to the online survey. This test ensured that the survey features, such as question randomisation, skip logic, and answer recording, were functioning correctly and consistently. Consultants participation was essential to highlight and resolve any issues with the translation and wording of the questions, both for country-specific surveys and for those we fielded in multiple geographies to allow for cross-country comparisons. In collaboration with YouGov, we also conducted extensive pre-testing to ensure that all the survey questions were appropriately displayed, particularly for experiments containing complex randomisation of information or response options displayed or those question modules which employed imagery or large amounts of text.

Once the research team and consultants approved the questionnaire, we soft-launched the surveys collecting small samples of around 100 respondents. In these pilots, we checked the numeric codes in the data to ensure they matched the numeric values on the survey. We performed outlier and cross-tabulation analysis to find suggestive evidence of leading/biased questions in the survey. We also checked that survey logic testing worked, including checks that skip logic, branching, and other automated features worked as intended and were reflected in the data. We implemented a third quality control stage by obtaining and inspecting partial samples throughout data collection. These larger sets underwent the same quality controls undertaken during the pilot testing described above. We also extended our inspection by computing the proportion of respondents who systematically answered ’Don’t know/Prefer not to say’ as a signal of low engagement or survey fatigue. We also checked for unusual skewness in the respondents’ answers. A final set of similar tests were conducted on the final dataset once all data collection was completed.

Further to our quality control strategies, YouGov also employed a robust set of quality assurance measures to ensure the reliability and validity of survey responses. These measures were implemented prior to the full data being delivered to us. During data collection, respondents were subjected to checks for attentiveness, including attention checks that explicitly require respondents to select a specific answer and validations of demographic responses against pre-existing panel data to flag inconsistencies. Additionally, speeders (respondents who complete surveys significantly faster than the median time) and straight-liners (those who select top answer option for each question) were identified and removed from the sample. Further quality control processes included verifying IP addresses, geo-location information, and screening for overactive IPs to prevent fraudulent or duplicate participation. The cleaning process also included monitoring item non-response and identifying patterns in ‘Don’t know’ responses to ensure the data were robust. Respondents flagged through these checks were excluded from the final dataset. On average, this led to the exclusion of 17.1% of respondents in Tracker surveys, 2.8% in Panel surveys, and 4% in Sandbox surveys in the most recent surveys at the time of writing. The higher retention in panel surveys reflects the recontact nature of these samples and the typically higher engagement of respondents.

Note that we do not exclude any further responses from this dataset. Researchers can set their post-hoc exclusion approach(es) when they perform their analysis. The datasets contain information to exclude responses based on properties of responses, such as excluding answers with highly unusual values or the properties of producing the responses (e.g., survey completion times).

Lastly, the following steps were taken to ensure respondents’ anonymity. Respondent data are stored against a unique identifier assigned to each panel member, which allows us to match back to demographic information each time without storing the datasets together. We also grouped or binned variables such as income and place of residence into larger categories. Income is reported into income brackets, whereas variables such as place of residence are reported as administrative regions. For example, in Germany, the place of residence is categorised based on Nielsen’s micro-regions, whereas in Great Britain, the residence is recorded using the largest administrative geographical areas. Throughout the questionnaire design, we ensured not to elicit sensitive or personal information that would compromise individuals’ anonymity.

### Post-Harmonisation

We recoded the original data to homogenise their structure across the different types of datasets. Specifically, we standardised the numeric codes for 5-point and 10-point Likert scale items. We also homogenised the naming conventions used in the datasets, so researchers can easily merge the datasets in such a way that all variables follow the following structure: *variablename_wave* or *variablename_wave_number*. We inspected all codebooks, questionnaires, and data tables, ensuring this documentation matched with the datasets.

### R Data Package

We created an R Package called *DELdata* to facilitate access to the datasets. Researchers using data can easily load datasets into an R session using very few commands. To facilitate usage, we include a vignette that explains how researchers can load the data into an R session. The vignette is available at https://ftraposo.github.io/DELdata/docs/reference/get_del_data.html We also made additional documentation of this package available through GitHub: https://github.com/ftraposo/DELdata/tree/gh-pages^43^.

## Usage Notes

### Academic Usage

The benefits of longitudinal data will allow scholars to unlock new modelling approaches using dynamic data. A more comprehensive set of questions is essential to unlocking factor/latent class analysis approaches to capture constructs better. The current literature on public engagement with development and aid has been the lack of robust measures of public support for foreign aid^44^, which would allow for accurate estimates of latent public opinion. These data will enable departing the existing single-question approach and could provide a more efficient and less biased estimate of public support for foreign aid.

Country-level indexes have been widely used to compare countries in specific dimensions such as attitudes towards corruption, democracy, and governance. DEL data and surveys can serve as templates for researchers interested in constructing a country-level foreign aid support index. With these new data available, researchers could use the numerous items included in the 91 surveys to construct new forms of these indexes in the future.

### Pedagogical Usage

Given its depth and structure, the datasets can also be an important pedagogical tool. Instructors of quantitative methods courses can use the data to teach introductory concepts such as regression analysis and hypothesis testing or more advanced methodologies such as panel data estimation or conjoint experiments. The data also allow researchers to conduct multivariate analyses. Instructors can teach students the different methods to measure unobservable underlying traits, including principal component analysis and item response theory, as the data contains several sets of items that aim to capture specific constructs such as moral obligation, social norms, and perceptions of personal efficacy or aid effectiveness.

### NGO Usage

As DEL project stakeholders, international development NGOs have relied on these data to gain insight into the evolution of public attitudes and values around global poverty, development, and international cooperation, and the factors that shape these attitudes. Users from the third sector can use these data and insights to improve advocacy, messaging, and communications strategies. In particular, these insights can provide evidence on the frames, messages, and messengers that resonate or are more persuasive for donor publics. Our analysis of messengers is an example of where the data have proved particularly useful for NGOs and development agencies^45^.

In Table 15, we build upon our attrition analysis by regressing attrition status on the demographic characteristics collapsed by country rather than waves. Based on this analysis, attrition patterns are similar across countries, but there are some slight differences. Overall, we find that the attriters are younger than the observed sample. Regarding the proportion of women in the samples, we do not find significant differences across most countries except the United States. We expand on this attrition analysis in the appendix. In Table 16, we conducted the same analysis conducted in 15, but collapsing by wave. Across these analyses, the patterns of attrition point in the same direction: younger respondents with lower levels of education are more likely to withdraw from subsequent waves.

### Question Wording

#### Figure 1

Below is an illustration of the conjoint experiment presented in Fig. 1. The full list of attributes are illustrated in Fig. 1.

Thinking about development NGOs/charities that work to reduce poverty in poor countries, we are now going to present to you two NGOs. Please indicate which of the following NGOs/charities you would be more likely to donate to if asked.

Which of the following NGOs/charities are you more likely to donate to?

**Table:** 

**Development NGO/Charity A**	**Development NGO/Charity B**
Small NGO	Large NGO
Headquarters in the UK	Headquarters in a poor country
Sub-Saharan Africa	Latin America
Education	Water, sanitation, and hygiene

<1>Development NGO/Charity A

<2> Development NGO/Charity B

#### Figure 2

Below is the wording to measure ’support for foreign aid’ as illustrated in Fig. 2. The version of the question comes from GB Panel Wave 3 (2021) and the numbers and terminology are adapted by year and country.

[track7_w3]single Of its total budget of nearly €1,100 billion, the UK government currently allocates 1 percent, or €10.9 billion, to overseas aid to poor countries. Do you think that the government should increase or decrease the amount of money that it spends on overseas aid to poor countries?

- <1>Increase a great deal
- <2>Increase somewhat
- <3>Stay the same
- <4>Decrease somewhat
- <5>Decrease a great deal
- <6>Don’t know

## Supplementary information

Supplementary Information

## Data Availability

We make the data available in a readily accessible format along with the R scripts used to clean the data in DEL’s dataverse. These R code files include code to check that values fall within expected ranges and standardising variable names and values.^46^ We also made an annotated R script available to create a behavioural segmentation variable DELseg. This script allows researchers to replicate the segmentation created by the DEL team.^47^ This variable aims to capture different forms of engagement with development issues. A taxonomy diagram of this segmentation is provided alongside the script.
