# Supplementary material for: Survey Data on Attitudes Towards Foreign Aid & Development in France, Germany, Great Britain, and the U.S
Source: Sci Data. 2025 Jul 1;12:1122. doi: 10.1038/s41597-025-05135-0 (PMC12217255; doi:10.1038/s41597-025-05135-0)
Supplement: Supplementary file 1 — Supplementary Information [file 41597_2025_5135_MOESM1_ESM.pdf]

Appendix

Tables

Table 2. Summary Data Collection

Country	Year/Periods	Number of surveys	Frequency (per year)	Sample
A. Panel				
France	2019 - 2024	6	1	6001 to 6073
Germany	2019 - 2024	6	1	6000 to 6050
Great Britain	2019 - 2024	6	1	8008 to 8281
United States	2019 - 2024	6	1	6004 to 6112
B. Trackers				
France	2020 - 2024	10	2	1000 to 1081
Germany	2020 - 2024	10	2	1001 to 1141
Great Britain	2020 - 2024	10	2	1036 to 2000
United States	2020 - 2024	10	2	1153 to 1343
C. Sandboxes				
France	2019 - 2023	9	1 (2019); 2 (2020-2023)	2000 to 2138
Germany	2020 - 2023	7	1 (2021); 2 (2020, 2022, 2023)	2002 to 3538
Great Britain	2020 - 2023	6	2	1761 to 3639
United States	2020 - 2023	5	2 (2020); 1 (2021); 2 (2023)	2009 to 3527

Note This table provides details of the data collection, sample size, and frequency of the three types of data collected throughout the Development Engagement Lab project. For Sandboxes, the periodicity of data collection varied by country and year. Given that the periodicity of Sandboxes varied year by year, we report the number of these surveys and the year they were conducted in parenthesis. No sandbox was conducted in 2024.

Table 3. Multi-group Confirmatory Factor Analysis by Country and Wave - Selected Items

Model	χ^2	(df)	CFI	RMSEA (90% CI)	SRMR
**By Country**					
Configural	1247.704	20	0.975	0.092	0.031
Metric	1593.517	32	0.969	0.082	0.047
Scalar	3480.409	44	0.931	0.104	0.063
Strict	4842.825	59	0.904	0.105	0.081
**By Wave**					
Configural	1066.943	40	0.979	0.084	0.030
Metric	1126.097	68	0.978	0.065	0.032
Scalar	1331.656	96	0.974	0.059	0.036
Strict	1433.891	131	0.973	0.052	0.038

Note: This table reports the Multi-group Confirmatory Factor Analysis using six core items in both *Panel* and *Trackers*. The analysis only contains *Tracker* data from all four countries and for all eight waves, totalling 29,158 observations. The analysis was conducted by collapsing observations by *Country* and by *Country*. We excluded all missing values from the analysis from Do not know/Prefer not to say.

Table 4. Summary of questions in Panel

Area	Number of questions
Track	10
Engagement	4
Charitable donations	2-4
Core Aid	3
Economy	3
Costs and benefits	4
Morality	4
Social norms	3
Trust	3
Efficacy	2
Democracy and Partisanship	6
Immigration	4
Sustainable Development Goals	1
Other issues	4

Note: This table reports the structure of the survey used for *Panel*. It summarises the number of topics covered and the number of questions asked in each battery.

Table 5. Details Panel Data

Country	Year	Collection Period	Sample	Sample retention	Retention rate (%)
France	2019	27 September to 19 October	6073	-	-
	2020	21 September to 12 October	6001	4173	69
	2021	24 September to 22 October	6106	3603	59
	2022	30 September to 4 November	6051	2668	44
	2023	15 September to 23 October	6028	2211	36
	2024	26 September to 29 October	6010	2278	38
Germany	2019	24 September to 10 October	6004	-	
	2020	10 September to 13 October	6000	4296	72
	2021	22 September to 22 October	6000	3733	62
	2022	30 September to 3 November	6008	2665	44
	2023	19 September to 23 October	6050	2189	36
	2024	3 October to 30 October	6048	2204	37
Great Britain	2019	18 September to 10 October	8037	-	-
	2020	10 September to 12 October	8079	5932	74
	2021	23 September to 25 October	8281	4456	55
	2022	30 September to 28 October	8008	3880	48
	2023	15 September to 18 October	8018	3680	46
	2024	11 October to 6 November	8090	3399	42
United States	2019	20 September to 9 October	6004	-	-
	2020	11 September to 18 October	6018	3811	63
	2021	23 September to 27 October	6112	2680	45
	2022	30 September to 24 October	6102	2508	42
	2023	15 September to 16 October	6095	2178	36
	2024	1 October to 30 October	6032	1994	33

Note: This table reports the collection times, sample size, retention rates, and retention samples of *Panel* for all countries from the first round in 2019 until the latest panel collected in 2024.

Table 6. Coverage of topics covered across panel surveys

	France						Germany						Great Britain						United States					
	W1	W2	W3	W4	W5	W6	W1	W2	W3	W4	W5	W6	W1	W2	W3	W4	W5	W6	W1	W2	W3	W4	W5	W6
Charities/NGO attitudes																								
Climate & environment																								
Conflict																								
Development aid																								
Donations																								
Education																								
Efficacy																								
Humanitarian aid																								
Gender																								
Global health																								
Imagery																								
Inequality																								
Media																								
Migration & Refugees																								
Poverty																								
Sustainable Development Goals																								
Trust																								

Note: This table provides information about the coverage of the topics covered in *Panel*. Red represents no coverage. Green represents coverage of that topic. Blue represents extensive coverage.

Table 7. Retention rates Wave by Wave (%)

Waves	France	Germany	Great Britain	United States
Wave 1 - Wave 2	69	72	74	63
Wave 2 - Wave 3	72	68	65	52
Wave 3 - Wave 4	62	63	65	65
Wave 4 - Wave 5	65	63	69	61
Wave 5 - Wave 6	70	64	67	56

Note: This table reports all countries' retention rates for subsequent waves. We calculated retention rates by collapsing the data by waves without disgregating by country.

Table 8. Demographics - Panels

**Proportion of Women**						
Country	2019<br>Wave 1	2020<br>Wave 2	2021<br>Wave 3	2022<br>Wave 4	2023<br>Wave 5	2024<br>Wave 6
France	0.52	0.52	0.51	0.51	0.51	0.52
	(0.01)	(0.01)	(0.01)	(0.01)	(0.01)	(0.01)
Germany	0.52	0.51	0.51	0.52	0.52	0.52
	(0.01)	(0.01)	(0.01)	(0.01)	(0.01)	(0.01)
Great Britain	0.52	0.51	0.52	0.52	0.52	0.52
	(0.01)	(0.01)	(0.01)	(0.01)	(0.01)	(0.01)
United States	0.52	0.51	0.51	0.51	0.52	0.52
	(0.01)	(0.01)	(0.01)	(0.01)	(0.01)	(0.01)
**Age (Yrs)**						
France	48	49	50	32	48	48
	(0.23)	(0.34)	(0.27)	(0.21)	(0.27)	(0.27)
Germany	49	50	50	49	48	48
	(0.27)	(0.37)	(0.27)	(0.19)	(0.27)	(0.25)
Great Britain	31	50	48	49	48	48
	(0.28)	(0.28)	(0.21)	(0.2)	(0.25)	(0.25)
United States	49	33	32	48	48	48
	(0.3)	(0.28)	(0.22)	(0.29)	(0.25)	(0.24)
**Partisanship (0 to 10 scale)**						
France	7	7	6	8	6	6
	(0.05)	(0.06)	(0.04)	(0.04)	(0.05)	(0.05)
Germany	7	6	6	6	6	6
	(0.05)	(0.05)	(0.04)	(0.04)	(0.05)	(0.05)
Great Britain	8	6	7	6	6	6
	(0.05)	(0.04)	(0.04)	(0.04)	(0.05)	(0.05)
United States	6	7	8	6	6	6
	(0.05)	(0.04)	(0.04)	(0.06)	(0.05)	(0.05)
**Monthly income ($USD)**						
France	2988	3022	2979	3075	3145	3310
	(32)	(34)	(34)	(36)	(66)	(44)
Germany	2861	2921	3007	3065	3233	3424
	(37)	(33)	(32)	(35)	(34)	(36)
Great Britain	4001	4093	4301	4369	4659	4912
	(44)	(46)	(46)	(46)	(49)	(52)
United States	5648	5281	5776	6225	5906	6306
	(122)	(96)	(109)	(118)	(98)	(106)

Note: This table summarises the main covariates for all five Panel waves. The "Proportion of Women" category indicates the percentage of respondents who identified as women. "Age" refers to the age of the respondents at the time of the survey. "Partisanship" is based on a self-reported scale ranging from 0 to 10. The monthly household income covariate is reported in USD for all four countries. For respondents in France and Germany, we used a conversion rate of 1.11 USD. For Great Britain, the conversion rate used was 1.32 USD. Survey weights were applied to ensure that the estimates were nationally representative. Standard errors are reported in parenthesis.

Table 9. Attrition analysis - Covariates

Covariates	Wave 1	Wave 2		Wave 3		Wave 4		Wave 5		Wave 6		Completion types	
	Baseline wave	Observed	Attriters	Observed	Attriters	Observed	Attriters	Observed	Attriters	Observed	Attriters	Partial completers	Completers
Age (Yrs)	46.94	51.68	51.68	52.67	43.52	53.45	44.63	53.66	45.30	54.70	44.87	49.55	52.29
	(18.07)	(15.93)	(15.93)	(15.42)	(17.43)	(14.95)	(17.48)	(14.80)	(17.46)	(14.28)	(17.39)	(16.70)	(15.54)
Proportion of Women	0.46	0.46	0.46	0.46	0.45	0.47	0.45	0.47	0.45	0.46	0.45	0.44	0.45
	(0.50)	(0.50)	(0.50)	(0.50)	(0.50)	(0.50)	(0.50)	(0.50)	(0.50)	(0.50)	(0.50)	(0.50)	(0.50)
Education (Tertiary)	0.39	0.30	0.30	0.29	0.30	0.31	0.28	0.31	0.28	0.41	0.40	0.28	0.31
	(0.49)	(0.46)	(0.46)	(0.45)	(0.46)	(0.46)	(0.45)	(0.46)	(0.45)	(0.49)	(0.49)	(0.45)	(0.46)
Partisanship (1 to 10)	5.57	5.11	5.11	5.13	5.01	5.17	5.01	5.20	5.00	5.20	5.00	4.97	5.12
	(2.90)	(2.59)	(2.59)	(2.56)	(2.66)	(2.58)	(2.63)	(2.58)	(2.62)	2.58	2.62	(2.63)	(2.56)
Income ($USD)	4125.31	3841.19	3841.19	3727.77	4186.78	3869.09	3976.01	3917.92	3932.02	3894.10	3946.85	3754.20	3756.29
	(3994.95)	(3503.63)	(3503.63)	(3355.03)	(4515.07)	(3516.76)	(4214.43)	(3512.57)	(4153.21)	(3460.20)	(4165.45)	(3604.85)	(3482.72)

Note: This table provides summary statistics for observed samples and attriters. Age is measured in years, and "Proportion of Women" is binary equal to 1 if the respondent reported their gender as "Women". Education is binary equal to 1 if education is college education or above. We excluded Germany for this variable, as we only contain primary and secondary educational attainment for this country. Partisanship is measured from 0 (Left) to 10 (Right). Income is measured as the average income using the average of each of the income brackets in each country and each year. Estimates were weighted to attain nationally representative samples.

Table 10. Details Tracker Data

Country	Year	Collection period	Sample
France	2020	9 to 13 January	1003
	2020	3 to 7 June	1000
	2021	6 to 8 Jan	1060
	2021	7 to 9 June	1081
	2022	14 to 17 Jan	1055
	2022	1 to 2 June	1005
	2023	4 to 10 January	1081
	2023	30 May to 2 June	1003
	2024	8 to 15 January 2024	1020
	2024	3 to 9 June 2024	1028
Germany	2020	9 to 16 January	1141
	2020	3 to 7 June	1025
	2021	6 to 7 Jan	1004
	2021	2 to 6 June	1001
	2022	13 to 14 Jan	1015
	2022	26 May to 6 June	1038
	2023	3 to 9 Jan	1100
	2023	30 May to 2 June	1003
	2024	8 to 13 January 2024	1021
	2024	3 to 7 June 2024	1004
Great Britain	2020	9 to 13 January	1036
	2020	3 to 4 June	1705
	2021	5 to 6 Jan	1708
	2021	2 to 3 June	1703
	2022	12 to 13 Jan	1690
	2022	30 May to 1 June	2082
	2023	5 to 6 January	1693
	2023	30 to 31 May	2000
	2024	8 to 9 January 2024	2031
	2024	4 to 5 June 2024	2147
United States	2020	8 to 13 January	1153
	2020	3 to 4 June	1343
	2021	6 to 7 Jan	1207
	2021	4 to 9 June	1227
	2022	12 to 13 Jan	1240
	2022	27 to 31 May	1237
	2023	4 to 5 Jan	1163
	2023	30 to 31 May	1326
	2024	9 to 10 January 2024	1164
	2024	3 to 4 June 2024	1208

Note: This table reports the collection times and sample sizes of *Trackers* for all countries from the first cross-section 2019 until the last round in 2024.

Table 11. Demographics - Trackers

Country	Wave 1	Wave 2	Wave 3	Wave 4	Wave 5	Wave 6	Wave 7	Wave 8	Wave 9	Wave 10
**Proportion of Women**										
France	0.52	0.52	0.52	0.52	0.52	0.52	0.52	0.52	0.52	0.52
	(0.02)	(0.02)	(0.01)	(0.01)	(0.01)	(0.01)	(0.01)	(0.01)	(0.01)	(0.01)
Germany	0.51	0.51	0.51	0.51	0.51	0.51	0.50	0.51	0.51	0.51
	(0.01)	(0.01)	(0.01)	(0.01)	(0.01)	(0.01)	(0.01)	(0.01)	(0.01)	(0.01)
Great Britain	0.52	0.51	0.51	0.51	0.51	0.51	0.52	0.52	0.52	0.52
	(0.01)	(0.01)	(0.01)	(0.01)	(0.01)	(0.01)	(0.01)	(0.01)	(0.01)	(0.01)
United States	0.51	0.51	0.51	0.52	0.52	0.52	0.52	0.52	0.52	0.52
	(0.01)	(0.01)	(0.01)	(0.01)	(0.01)	(0.01)	(0.01)	(0.01)	(0.01)	(0.01)
**Age (Yrs)**										
France	48	48	49	49	50	50	50	49	50	49
	(0.57)	(0.79)	(0.56)	(0.59)	(0.58)	(0.56)	(0.59)	(0.64)	(0.83)	(1.03)
Germany	50	50	50	50	50	50	50	50	50	50
	(0.54)	(0.66)	(0.69)	(0.72)	(0.7)	(0.83)	(0.68)	(0.61)	(0.61)	(0.59)
Great Britain	49	49	49	49	49	49	49	50	49	48
	(0.62)	(0.43)	(0.48)	(0.45)	(0.49)	(0.46)	(0.44)	(0.45)	(0.42)	(0.43)
United States	48	47	48	48	48	49	48	48	48	48
	(0.65)	(0.62)	(0.58)	(0.54)	(0.61)	(0.57)	(0.65)	(0.61)	(0.62)	(0.57)
**Monthly income ($USD)**										
France	3062	3052	3067	2943	2930	2926	2957	3092	1618	3083
	(99)	(80)	(78)	(79)	(68)	(77)	(78)	(117)	(95)	(95)
Germany	2941	2967	2807	2887	2832	3054	3046	2931	3214	3534
	(74)	(86)	(75)	(67)	(66)	(78)	(76)	(77)	(86)	(113)
Great Britain	4409	4274	4428	4383	4555	4364	4846	4878	5259	5256
	(157)	(103)	(107)	(98)	(107)	(98)	(112)	(118)	(106)	(100)
United States	5591	5562	5313	5668	5765	5544	5910	6065	5808	6071
	(253)	(180)	(178)	(222)	(225)	(213)	(223)	(236)	(208)	(210)

Note: This table summarises the main covariates for all Panel waves. The "Proportion of Women" category indicates the percentage of respondents who identified as women. "Age" refers to the age of the respondents at the time of the survey. "Partisanship" is based on a self-reported scale ranging from 0 (Left) to 10 (Right). The monthly household income covariate is reported in USD for all four countries. For respondents in France and Germany, we used a conversion rate of 1.11 USD. For Great Britain, the conversion rate used was 1.32 USD. Survey weights were applied to ensure that the estimates were nationally representative. Standard errors are reported in parenthesis.

Table 12. Details Sandboxes Data

Country	Year	Collection period	Sample
France	2019	8 to 30 May 2019	2138
	2020	5 to 13 February	2003
	2020	21 to 28 July	2042
	2021	30 April to 5 May	2066
	2021	5 to 10 August	2001
	2022	13 to 21 Jan	2017
	2022	7 to 15 June	2070
	2023	20 Feb to 1 Mar	2046
	2023	19 to 25 May	2000
Germany	2020	7 to 14 February	2009
	2020	15 to 23 July	2012
	2021	28 July to 3 Aug	2010
	2022	11 to 23 Mar	2036
	2022	22 to 29 June	2059
	2023	17 to 28 Feb	2002
	2023	10 to 16 October	3538
Great Britain	2020	26 to 17 Apr	1761
	2020	7 to 13 July	2009
	2021	11 to 18 June	3023
	2022	23 to 28 June	2187
	2023	20 to 28 Feb	2007
	2023	9 to 18 Oct	3639
United States	2020	28 May to 1st June	2425
	2020	10 to 14 July	2009
	2021	6 to 22 Sep	5591
	2023	21 to 24 Feb	2345
	2023	11 to 16 October	3527

Note: This table reports the collection times and sample size of *Sandboxes* for all countries from the first round in 2019 until the last round in 2023. We did not conduct any *Sandboxes* in 2024.

Table 13. Topics covered across sandbox surveys

Country	Year	Month	Topics
France	2019	May	Role of France; Feminist Development Policy; Gender equality; Development Finance; Health; Deservingness
	2020	February	Gender equality
	2020	July	Knowledge; Information; Misinformation; SDGs; Cooperation; Trust
	2021	May	Finance; Africa; Challenges; Outlook; COVID; Cooperation; Climate Change; Corruption; International ;Organisations
	2021	August	COVID; Migration; Climate; Change (Energy production; transportation; Impact); Paris Agreement; Vaccines
	2022	Jan	COVID; Cooperation; Immigration/Migration; Climate Change (Health/Well-being; Taxation); Gender Equality; Women's Leadership
	2022	June	Cooperation; Refugees; Africa; Terminology; France Role
	2023	Mar	Feminist Development Policy; Gender Equality; Aid allocation; Cooperation; Knowledge; Citizenship
	2023	May	Imagery; Donations
Great Britain	2020	Apr	COVID; Messenger Testing
	2020	July	Knowledge; Information; Misinformation; SDGs; Cooperation; Trust
	2021	June	Video/Images and donations; Budget allocation
	2022	June	Ukraine; Refugees; Feminist Development Policy
	2023	Feb	Gender equality; Feminist Development Policy
	2023	Oct	Imagery; Donations
Germany	2020	February	Sustainable consumption; human rights; gender equality
	2020	July	Knowledge; Information; Misinformation; SDGs; Cooperation; Trust
	2021	Aug	Development Priorities; Climate Change (CC and development, investments in CC mitigation; gov & personal actions on CC); COVID-19 (Vaccine equity); SDGs (Knowledge; perceptions of achievement; trade-off)
	2022	Mar	Priorities in government spending; G7; Afghanistan; Climate Change; Vaccine equity
	2022	June	Crises; Funding; 0.7%; Terminology around "poor" countries; Ukraine; Cost of living crisis; Deservingness; Feminist Development Policy
	2023	Feb	Feminist Development Policy; Gender inequality; Women's policies; NGO types and donations
	2023	October	Imagery; Donations
United States	2020	June	Information & misinformation; SDGs; Cooperation & trust
	2020	July	COVID-19
	2021	Sep	Climate finance; Aid allocation; Synergy between issue areas; America's place in the world; Message testing
	2023	Feb	Feminist Development Policy; Gender inequality; Women's policies
	2023	October	Imagery; Donations

Note: This table reports the topics covered across sandboxes by country, year, and month. The themes are based on the batteries of questions included in each survey.

Table 14. Topics covered across tracker surveys

Country	Topics
France	Areas of concern, SDGs, sense of connectedness, donation, concern for poverty and development, attitudes on aid, efficacy, refugees, battery of engagement
Great Britain	Areas of concern, perception of state of the world, sense of connectedness, donation, concern for poverty and development, attitudes on aid, efficacy, trust in NGOs, battery of engagement
Germany	Areas of concern, donation, concern for poverty and development, arguments for aid, attitudes on aid, efficacy, trust in NGOs, battery of engagement
United States	Areas of concern, perception on state of the world, sense of connectedness, donation, concern for poverty and development, attitudes on aid, efficacy, trust in NGOs

Note: This table shows the topics covered across trackers. While we captured the same set of items across the different waves and countries, we expanded our questionnaire for some waves/countries to elicit beliefs, attitudes, and behaviours regarding a more extensive set of development issues.

In Table 15, we build upon our attrition analysis by regressing attrition status on the demographic characteristics collapsed by country rather than waves. Based on this analysis, attrition patterns are similar across countries, but there are some slight differences. Overall, we find that the attriters are younger than the observed sample. Regarding the proportion of women in the samples, we do not find significant differences across most countries except the United States. We expand on this attrition analysis in the appendix. In Table 16, we conducted the same analysis conducted in Table 15, but collapsing by wave. Across these analyses, the patterns of attrition point in the same direction: younger respondents with lower levels of education are more likely to withdraw from subsequent waves.

Table 15. Attrition Analysis by Country

	France	Germany	Great Britain	United States
Intercept	0.753***	0.876***	0.604***	0.891***
	(0.042)	(0.086)	(0.039)	(0.036)
Age	-0.007***	-0.004***	-0.005***	-0.007***
	(0.000)	(0.000)	(0.000)	(0.000)
Proportion of Women	-0.001	-0.021*	0.008	0.038***
	(0.007)	(0.010)	(0.007)	(0.008)
Education (Tertiary)	-0.013+		-0.026***	-0.030***
	(0.008)		(0.007)	(0.009)
Partisanship (0 to 10)	-0.002	0.001	-0.002	0.000
	(0.001)	(0.003)	(0.002)	(0.001)
Log Income	0.006	-0.010	0.010*	-0.011*
	(0.005)	(0.007)	(0.005)	(0.004)
Still education		0.013		
		(0.084)		
Low-secondary		-0.101		
		(0.068)		
Mid-secondary		-0.151*		
		(0.067)		
Top-secondary		-0.136*		
Num.Obs.	20980	17388	21735	22235
R2	0.053	0.019	0.036	0.063
R2 Adj.	0.053	0.018	0.035	0.063

Note: This table presents regression estimates for attrition analysis across countries. The dependent variable is attrition status. Attriters are coded as 1, whereas respondents who answered the survey are coded as 0. Age is measured in years, and "Proportion of Women" is binary equal to 1 if the respondent reported their gender as "Women". Education is binary equal to 1 if education is tertiary education or above. We separated Germany as the education variable as it contains education levels before university education. Standard errors are in parentheses. All regressions were weighted using survey weights to ensure nationally representative estimates.
*** $p < 0.001$, ** $p < 0.01$, * $p < 0.05$, + $p < 0.10$

Table 16. Attrition Analysis by Wave

Covariates	Wave 2		Wave 3		Wave 4		Wave 5		Wave 6	
	FR/GB/US	Germany	FR/GB/US	Germany	FR/GB/US	Germany	FR/GB/US	Germany	FR/GB/US	Germany
Intercept	0.690***	1.008***	0.724***	0.630***	0.966***	1.067***	0.991***	1.387***	1.064***	1.049***
	(0.047)	(0.136)	(0.049)	(0.107)	(0.048)	(0.147)	(0.046)	(0.123)	(0.043)	(0.141)
Age	-0.008***	-0.005***	-0.008***	-0.006***	-0.008***	-0.006***	-0.008***	-0.004***	-0.009**	-0.005***
	(0.000)	(0.001)	(0.000)	(0.001)	(0.000)	(0.001)	(0.000)	(0.001)	(0.000)	(0.001)
Female	0.019*	-0.005	0.005	0.009	0.013	-0.025	0.020*	-0.023	0.031***	-0.034+
	(0.009)	(0.018)	(0.009)	(0.019)	(0.009)	(0.020)	(0.009)	(0.020)	(0.009)	(0.020)
Education (Tertiary)	-0.070***		-0.012		-0.038***		-0.039***		-0.032***	
	(0.010)		(0.011)		(0.011)		(0.011)		(0.009)	
Log Income	0.001	-0.001	0.014*	0.012	-0.002	-0.016	-0.003	-0.043**	-0.003	-0.003
	(0.005)	(0.012)	(0.006)	(0.013)	(0.006)	(0.015)	(0.006)	(0.014)	(0.005)	(0.015)
Still education		-0.270+				0.047		-0.016		0.022
		(0.142)				(0.126)		(0.084)		(0.115)
Low-secondary		-0.452***				-0.113		-0.202**		-0.087
		(0.107)				(0.108)		(0.073)		(0.099)
Mid-secondary		-0.517***				-0.181+		-0.251***		-0.172+
		(0.106)				(0.107)		(0.097)		(0.097)
Top-secondary		-0.512***				-0.169		-0.236**		-0.138
		(0.106)				(0.107)		(0.072)		(0.097)
Partisanship (0 to 10)	0.003+	-0.004	0.002	0.000	0.001	0.005	-0.002	0.002	0.001	-0.002
	(0.002)	(0.005)	(0.002)	(0.005)	(0.002)	(0.005)	(0.002)	(0.005)	(0.002)	(0.005)
Num.Obs.	12990	4347	12990	4347	12990	4347	12990	4347	12990	4347
R2	0.097	0.046	0.074	0.053	0.083	0.038	0.070	0.031	0.096	0.034
R2 Adj.	0.097	0.044	0.074	0.051	0.083	0.036	0.069	0.029	0.096	0.032

Note: This table presents regression estimates for attrition analysis across waves. The dependent variable is attrition status. Attriters are coded as 1, whereas respondents who answered the survey are coded as 0. Age is measured in years, and "Proportion of Women" is binary equal to 1 if the respondent reported their gender as "Women". Education is binary equal to 1 if education is tertiary education or above. We separated Germany as the education variable as it contains education levels before university education. Standard errors are in parentheses. All regressions are weighted using survey weights to ensure nationally representative estimates. *** $p < 0.001$, ** $p < 0.01$, * $p < 0.05$, + $p < 0.10$

Question Wording

Figure 1

Below is an illustration of the conjoint experiment presented in Figure 1. The full list of attributes are illustrated in Figure 1.

Thinking about development NGOs/charities that work to reduce poverty in poor countries, we are now going to present to you two NGOs. Please indicate which of the following NGOs/charities you would be more likely to donate to if asked.

Which of the following NGOs/charities are you more likely to donate to?

**Development NGO/Charity A**	**Development NGO/Charity B**
Small NGO	Large NGO
Headquarters in the UK	Headquarters in a poor country
Sub-Saharan Africa	Latin America
Education	Water, sanitation, and hygiene

<1> Development NGO/Charity A
<2> Development NGO/Charity B

Figure 2

Below is the wording to measure 'support for foreign aid' as illustrated in Figure 2. The version of the question comes from GB Panel Wave 3 (2021) and the numbers and terminology are adapted by year and country.

`[track7_w3]{single}` Of its total budget of nearly £1,100 billion, the UK government currently allocates 1 percent, or £10.9 billion, to overseas aid to poor countries. Do you think that the government should increase or decrease the amount of money that it spends on overseas aid to poor countries?

<1>Increase a great deal
<2>Increase somewhat
<3>Stay the same
<4>Decrease somewhat
<5>Decrease a great deal
<6>Don't know
